# Population-Level Distribution, Risk Factors, and Burden of Mortality and Disability-Adjusted Life Years Attributable to Major Noncommunicable Diseases in Western Europe (1990-2021): Ecological Analysis

**DOI:** 10.2196/57840

**Published:** 2024-10-17

**Authors:** Sumaira Mubarik, Shafaq Naeem, Hui Shen, Rabia Mubarak, Lisha Luo, Syeda Rija Hussain, Eelko Hak, Chuanhua Yu, Xiaoxue Liu

**Affiliations:** 1 PharmacoTherapy, Epidemiology, and Economics, Groningen Research Institute of Pharmacy, University of Groningen, Groningen, Netherlands; 2 Department of Preventive Medicine, School of Public Health, Wuhan University, Wuhan, China; 3 Department of Communicable Disease Control and Prevention, Wuhan Center for Disease Control and Prevention, Wuhan, China; 4 Department of Economics, Arid Agriculture University, Rawalpindi, Pakistan; 5 Center for Evidence-Based and Translational Medicine, Zhongnan Hospital of Wuhan University, Wuhan, China; 6 Department of Medicine, Rawalpindi Medical University, Rawalpindi, Pakistan; 7 Department of Epidemiology and Biostatistics, School of Public Health, Wuhan University, Wuhan, China; 8 Global Health Research Division, Public Health Research Center and Department of Public Health and Preventive Medicine, Wuxi School of Medicine, Jiangnan University, Jiangsu, China

**Keywords:** mortality, smoking, Western Europe, CVDs, cardiovascular disease, HDI, Human Development Index, neoplasms, cancer, DALYs, disability-adjusted life years

## Abstract

**Background:**

Cardiovascular diseases (CVDs) and neoplasms are leading causes of mortality worldwide.

**Objective:**

This study aims to provide a comprehensive analysis of the mortality burden and disability-adjusted life years (DALYs) attributable to CVDs and neoplasms in Western Europe, investigate associated risk factors, and identify regional disparities. Additionally, the study evaluates the effectiveness of the Action Plan for the Prevention and Control of Non-Communicable Diseases (NCDs) in promoting healthier lives in the region.

**Methods:**

The study collected data on mortality and DALYs due to CVDs and cancers from 24 Western European countries using the Global Burden of Disease Study 2021. The analysis explored age, sex, and country-specific patterns, as well as risk factors contributing to these deaths. Additionally, the study examined time trends by calculating the annual percent change in mortality rates from 1990 to 2021 by region and cause.

**Results:**

In 2021, CVDs and neoplasms accounted for 27.8% and 27.1% of total deaths in Western Europe, with age-standardized death rates of 106.8 and 125.8 per 100,000, respectively. The top two CVDs in this region were ischemic heart disease and stroke, with age-standardized death rates of 47.27 (95% uncertainty interval [UI] 50.42-41.45) and 27.06 (95% UI 29.17-23.00), respectively. Similarly, the top two neoplasms were lung cancer and colorectal cancer, with age-standardized death rates of 26.4 (95% UI 27.69-24.47) and 15.1 (95% UI 16.25-13.53), respectively. Between 1990 and 2021, CVD mortality rates decreased by 61.9%, while cancer rates decreased by 28.27%. Finland had the highest CVD burden (39.5%), and Monaco had the highest rate of cancer-related deaths (34.8%). Gender differences were observed, with males experiencing a higher burden of both CVDs and cancer. Older individuals were also more at risk. Smoking had a stronger impact on CVD mortality and DALYs in males, while a higher Human Development Index was associated with increased cancer deaths and DALYs in females.

**Conclusions:**

The study findings highlight the substantial burden of NCDs, particularly CVDs and cancer, in Western Europe. This underscores the critical need for targeted interventions and effective implementation of the Action Plan for the Prevention and Control of NCDs to achieve the goal of ensuring healthy lives for all.

## Introduction

Noncommunicable diseases (NCDs), such as cardiovascular diseases (CVDs) and neoplasms (cancers), have become a significant global health challenge, leading to a substantial burden of morbidity and mortality [1]. In Western Europe, the prevalence of NCDs has been steadily increasing, with cancers and CVDs accounting for over 80% of disability-adjusted life years (DALYs) and more than 90% of fatalities [2]. Therefore, it is crucial to understand the distribution, risk factors, and death burden associated with these major NCDs to develop effective strategies for mitigating their impact on public health [3].

Globally, numerous strategies have been devised to address the prevention and control of NCDs [4]. A key initiative in this regard is the Action Plan for the Prevention and Control of NCDs in the WHO European Region 2016‐2025 [5]. It is a strategic plan developed by the World Health Organization (WHO) specifically for the European region. This plan offers a framework for countries in the region to create customized national strategies and action plans. It promotes collaboration, knowledge sharing, and capacity building to tackle shared challenges related to NCDs. The main goal is to improve health outcomes for their populations by implementing targeted measures and fostering cooperation among nations [6].

In Western Europe, cancer and CVDs are prominent NCDs and contribute significantly to premature mortality rates [7]. These 2 diseases have consistently been identified as leading causes of death and DALYs in Western Europe over the last 3 decades [8]. Therefore, in light of the escalating burden of NCDs in Western Europe and the global emphasis on addressing NCDs [1], this study aims to comprehensively investigate the distribution, risk factors, and death burden associated with CVDs and neoplasms in Western Europe from 1990 to 2021. Additionally, it seeks to evaluate the extent to which the Action Plan for the Prevention and Control of NCDs has been successful in achieving its goal of ensuring healthy lives for all.

By examining data on deaths and DALYs, the study offers updated insights into the death burden and impact of these major NCDs in Western Europe. Additionally, it explores the correlation between countries’ socioeconomic status, measured by the Human Development Index (HDI), and the burden of CVDs and neoplasms. The findings will provide valuable guidance for policy makers, health care professionals, and researchers in developing targeted interventions and policies to reduce the burden of NCDs, ultimately supporting the achievement of the Action Plan for the Prevention and Control of NCDs and enhancing population health outcomes.

## Methods

### Data Sources and Study Variables

The data on CVDs and neoplasms used in this study were obtained from the Global Burden of Disease Study 2021 (GBD 2021) database, which is publicly available on the VizHub - GBD Results platform [9]. This database is a comprehensive, collaborative effort coordinated by the Institute for Health Metrics and Evaluation at the University of Washington in the United States [8].

In this study, we extracted data on deaths and DALYs related to CVDs and neoplasms for the year 2021, stratified by gender in Western European countries. The data include the percentage of total deaths, age-standardized deaths, and age-specific deaths, spanning age groups from 20‐24 years to ≥95 years in 5-year intervals, along with 95% uncertainty intervals (UIs).

Furthermore, we analyzed the correlation between the socioeconomic status of countries and the burden of CVDs and neoplasms (deaths and DALYs) using the HDI. The HDI, sourced from the United Nations Human Development Report [10], assesses achievements in income, life expectancy, and education across countries [3].

### Ethical Considerations

The data used in this study were obtained from the GBD study, which provides publicly available, deidentified, and aggregated data. As such, no ethics board review or approval was required. According to our institutional policies and relevant guidelines, the use of publicly available and anonymized data does not require ethics approval.

### Statistical Analysis

A descriptive analysis was performed to examine the burden of CVDs and neoplasms across different age groups, gender (both sexes combined, male, and female), locations, and disease categories. The mortality burden was quantified as the percentage of total deaths and the age-standardized death rate per 100,000 population, including 95% UIs to account for statistical uncertainty.

### Age-Standardized Death Rates

The age-standardized death rates were calculated using the world standard population as defined by the GBD 2021 study. This method allows for the comparison of different populations or the same population over time, accounting for variations in age structures. The age-standardized death rates were reported per 100,000 population. The age-standardized death rate (ASR) calculation is given by the following equation:


$$ASR=\frac{\sum_{i=1}^{A}a_{i}w_{i}}{\sum_{i=1}^{A}w_{i}}\times 100,000$$

Where *a_i_* and *ω_i_* represent the age-specific rate and the number of persons (or weight) in the same age subgroup in the selected reference standard population.

### Time Trends

Time trends were assessed using the annual percent change (APC) from 1990 to 2021. A positive APC value indicates an increase in the burden of CVDs and neoplasms over the 32-year period, while a negative APC value suggests a decreasing trend. Uncertainty for each outcome was estimated using 1000 posterior distribution bootstrap samples, with UIs calculated from the 2.5th and 97.5th percentiles of these samples [11,12]. Point estimates and UIs were derived by taking the mean and the 25th and 975th values of the posterior distributions from the 1000 simulations. A 95% UI for the percentage change that did not include 0 indicated statistical significance.

This analysis included the age-standardized mortality rate for both males and females, as well as gender-specific APCs. Additionally, the population was divided into specific age groups (20‐24, 25‐29, 30‐34, 35‐39, 40‐44, 45‐49, 50‐54, 55‐59, 60‐64, 65‐69, 70‐74, 75‐79, 80‐84, 85‐89, 90‐94, and ≥95 years) to investigate the distribution of disease burden from CVDs and neoplasms by age in Western Europe.

### Correlation Analysis Between Covariates and Death and DALYs Across Different Quantiles

We used quantile regression to explore the associations between age-standardized rates of deaths and DALYs due to CVDs and neoplasms and several covariates, including the HDI, summary exposure value of smoking (SEV-Smoking), and summary exposure value of ambient particulate matter pollution (SEV-APMP) across 24 countries. The goal was to determine whether the impact of HDI, SEV-Smoking, and SEV-APMP on the burden of CVDs and neoplasms differs across various quantiles of the distribution.

Quantile regression was chosen for its ability to estimate the relationship between independent and dependent variables at different points in the distribution, providing a nuanced understanding of how covariates affect different quantiles of the outcome variable. The quantile regression estimates are based on minimizing the weighted absolute residuals, with the objective function defined as [13,14]:


$$min\left\{w_{t}\;\left|y_{t}-\alpha \right|\right\}=-\sum_{i:y_{i}<\alpha }^{T}\left(1-\tau \right)\left(y_{t}-\alpha \right)+\sum_{i:y_{i}\geq \alpha }^{T}\tau \left(y_{t}-\alpha \right)$$

where y_i_ represents the observed values, α is the quantile, and τ denotes the quantile level.

By utilizing quantile regression, we gained insights into how the effects of HDI, SEV-Smoking, and SEV-APMP on age-standardized death rates of CVDs and neoplasms vary across different segments of the population distribution. This approach offers a more comprehensive understanding compared to traditional linear regression models, which only estimate the average effect. All analyses and visualizations were performed using R software (version 4.3.2; R Foundation for Statistical Computing).

## Results

### Causes and Annual Trends of Disease Burden in Western Europe

**Figure 1.**
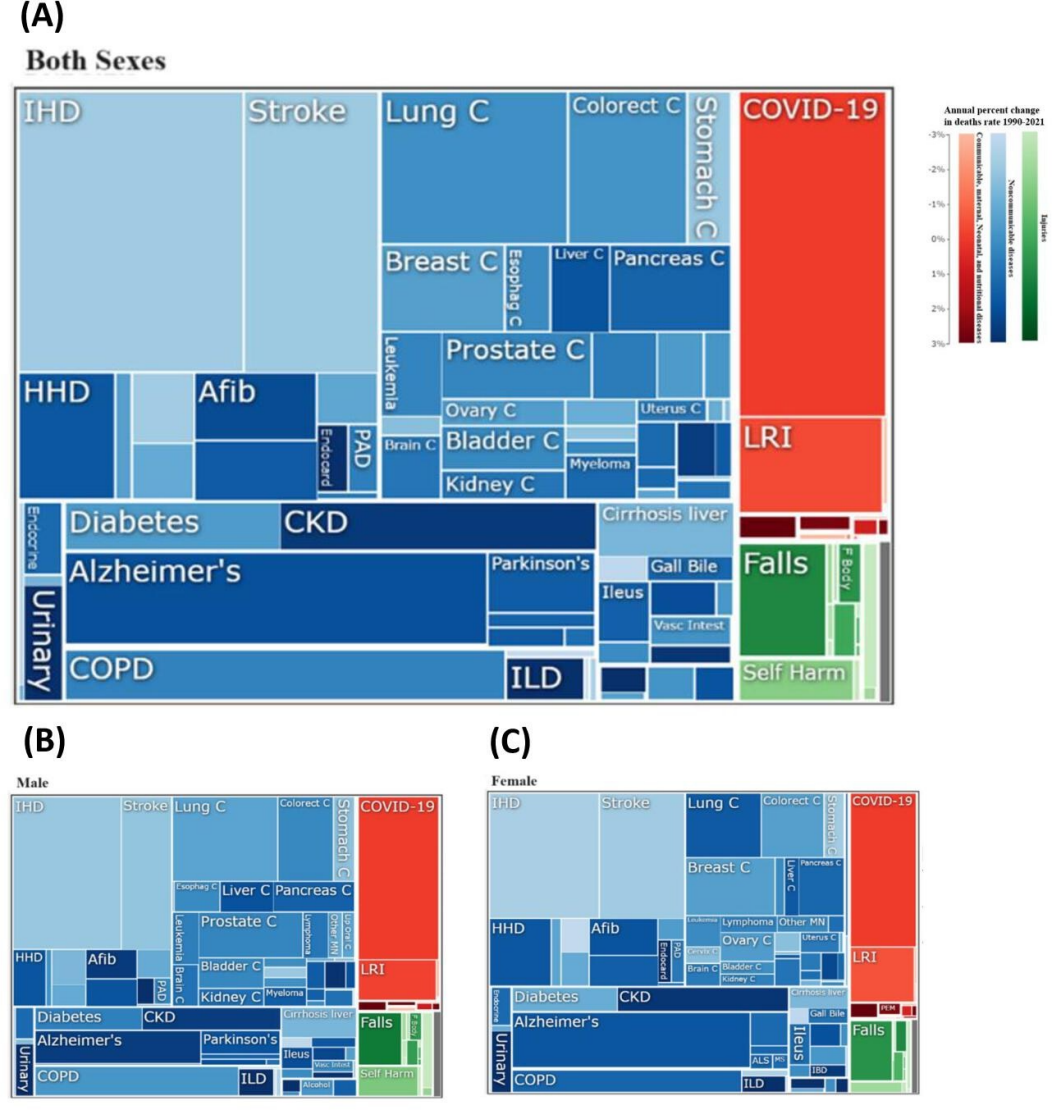
Causes of disease burden 2021 in all ages and annual percent change 1990‐2021 in age-standardized death rates (per 100,000) in Western Europe for (A) both sexes, (B) males, and (C) females. Box size indicates the percentage of all deaths, and color intensity or darkness indicates percent change 1990‐2021. Changes were calculated using the annual percent change formula 1990‐2021. Afib: atrial fibrillation and flutter; COPD: chronic obstructive pulmonary disease; CKD: chronic kidney disease; F Body: foreign body; HHD: hypertensive heart disease; IHD: ischemic heart disease; ILD: interstitial lung disease; LRI: lower respiratory infection; PAD: peripheral artery disease.

In 2021, following the COVID-19 pandemic, NCDs were the primary contributors to the burden of disease in Western Europe (Figure 1). Among the 11 most significant NCDs, CVDs and neoplasms were predominant, accounting for the highest death rates across all age groups in every Western European country in 2021 (Figure 1).

### CVD Statistics

In 2021, CVDs accounted for 27.8% (95% UI 30.0%-23.3%) of total deaths in Western Europe, with an age-standardized death rate of 106.8 (95% UI 114.4-91.8) per 100,000 population. The 3 leading subtypes of CVDs contributing to this mortality burden were ischemic heart disease, stroke, and ischemic stroke (Tables1  2). The death burden of CVDs in Western Europe has shown a downward trend from 1990 to 2021. The age-standardized death rate of CVDs in the region had a decrease of –61.9% (95% UI −60.7% to −64.3%) during this period. The decreases were particularly notable for the 3 leading subtypes of CVDs: ischemic heart disease (–67.2%, 95% UI −66.3% to −68.7%), stroke (–68.8%, 95% UI −67.7% to −70.2%), and ischemic stroke (–74.5%, 95% UI −73.5% to −75.9%) (Table 2).

**Table 1.** Burden of cardiovascular diseases and neoplasms across Western Europe, percent of total deaths in 2021 (95% UI^a^), all ages.

	Cardiovascular disease deaths (2021 estimate), % (95% UI)			Neoplasm deaths (2021 estimate), % (95% UI)		
	Both sexes	Male	Female	Both sexes	Male	Female
Western Europe	27.8 (30-23.3)	26.1 (27.3-23.4)	29.5 (32.8-23.2)	27.1 (28.6-24.3)	30.1 (31.2-27.8)	24.2 (25.9-20.8)
Andorra	24.7 (28.4-19.7)	18.4 (20.8-15.3)	33 (38.8-25.1)	30.8 (34.5-26.4)	37.1 (41.5-32.4)	22.6 (25.9-18.7)
Austria	32.9 (35.5-28)	30.3 (31.9-27.2)	35.5 (39.1-28.5)	24.2 (25.6-22)	26.3 (27.6-24.4)	22.2 (23.7-19.3)
Belgium	23.7 (25.9-19.7)	22.3 (23.7-19.9)	25.2 (28.4-19.6)	27.9 (29.7-25.1)	31 (32.7-28.6)	24.8 (26.9-21.3)
Cyprus	32.9 (35.6-29.3)	32.1 (34.4-29.2)	33.9 (37.4-29)	23.8 (25.6-21.6)	25.9 (28-23.6)	21.3 (23.4-18.9)
Denmark	22.9 (24.4-19.7)	22.9 (24.3-20.5)	22.8 (24.9-18.9)	32 (33.8-29.3)	33.4 (35.3-30.9)	30.5 (32.6-27.3)
Finland	39.5 (42.7-33)	37.9 (40.1-33.7)	41.1 (45.7-32.5)	25.1 (26.7-22.4)	26.2 (27.6-24)	24 (26-20.6)
France	23.8 (25.8-19.9)	21.3 (22.7-19.2)	26.2 (29.1-20.6)	30 (31.8-26.7)	34 (35.6-31.2)	25.9 (28.1-22.1)
Germany	31.6 (34.2-26.6)	30.1 (31.8-26.9)	33.2 (37.1-25.9)	25.6 (27.2-23)	27.9 (29.3-25.8)	23.2 (25.1-20.1)
Greece	33.5 (35.6-29)	30.2 (31.7-27.3)	37 (40.1-30.8)	24.7 (26.1-22.4)	28.5 (29.8-26.2)	20.8 (22.2-18.2)
Iceland	30.7 (33.3-25.7)	31.4 (33.4-27.5)	30 (33.5-23.8)	31.2 (33.1-27.9)	32.7 (34.8-29.9)	29.7 (31.9-25.9)
Ireland	25.7 (27.9-21.9)	25.9 (27.8-23.2)	25.5 (28.3-20.1)	28.6 (30.3-25.8)	29.5 (31.2-27.2)	27.6 (29.6-24.2)
Israel	21.1 (22.7-17.8)	20.6 (22-18.5)	21.6 (23.8-17.3)	27 (28.7-24)	27.8 (29.3-25.3)	26.2 (28.2-22.5)
Italy	30.9 (34-25.1)	28.3 (29.9-24.9)	33.5 (38-25.1)	27 (28.8-23.6)	31 (32.5-28.3)	23.2 (25.4-19.1)
Luxembourg	28 (29.9-24.8)	26.4 (27.8-24.4)	29.7 (32.2-24.9)	28.5 (30-26.3)	31.1 (32.7-29.1)	25.9 (27.5-23.2)
Malta	32.4 (34.9-27.4)	30.8 (32.7-27.4)	33.8 (37.2-27.4)	25.7 (27.2-23)	28.9 (30.7-26.6)	22.8 (24.4-19.9)
Monaco	25.4 (29.6-21.9)	23.4 (27.2-19.4)	27.5 (33.3-20.6)	34.8 (38.9-30.2)	37.2 (41.6-32.8)	32.2 (37-26.8)
Netherlands	23.8 (25.7-20.2)	22.9 (24.3-20.8)	24.7 (27.3-19.8)	29.4 (31-26.4)	32.5 (33.9-29.9)	26.3 (28.2-23.1)
Norway	26 (28.2-21.9)	25.2 (26.6-22.5)	26.8 (29.7-21.4)	30.4 (32.1-27.2)	33.2 (34.7-30.4)	27.8 (29.7-24)
Portugal	26.5 (28.6-22.5)	23.6 (24.9-21.6)	29.6 (32.7-23.6)	24.9 (26.4-22.3)	29.2 (30.7-27)	20.5 (22.2-17.5)
San Marino	27.4 (31.6-21.1)	23.7 (27.2-19.3)	31.7 (37.3-22.9)	28.1 (31.6-23.3)	32.4 (36.5-27.3)	23.2 (26.6-18.5)
Spain	25.9 (28.3-21.3)	23.4 (24.8-21.1)	28.5 (32.1-21.6)	26.5 (28.2-23.5)	31.4 (33.1-29)	21.3 (23.3-17.9)
Sweden	32.2 (34.8-27.2)	31.5 (33.3-28.1)	32.9 (36.3-26.3)	27.3 (29.2-24.4)	28.5 (30.2-25.9)	26.2 (28.4-22.6)
Switzerland	29.1 (32.1-23.6)	27.6 (29.4-24.2)	30.6 (34.8-22.9)	27.4 (29.3-24.1)	31.1 (32.7-28.6)	23.9 (26.1-19.9)
United Kingdom	23.3 (24.7-20.3)	23.3 (24.3-21.3)	23.3 (25.3-19.2)	27.3 (28.5-24.7)	28.2 (29.2-26.2)	26.4 (27.9-23.2)

a UI: uncertainty interval.

**Table 2.** Age-standardized deaths in 2021 (95% UI^a^) and annual percent change between 1990 and 2021 (95% UI) of the death rates in Western Europe.^b^

	Age-standardized deaths 2021 (95% UI)			Annual percent change 1990‐2021, % (95% UI)		
	Both sexes	Male	Female	Both sexes	Male	Female
**Cardiovascular disease**	106.8 (114.4 to 91.8)	132.4 (138.8 to 120.1)	85.4 (93.9 to 68.9)	−61.9 (−60.7 to −64.3)	−62.6 (−61.5 to −64.2)	−62.8 (−61.1 to −66.1)
Ischemic heart disease	47.27 (50.42 to 41.45)	66.83 (69.94 to 60.95)	31.7 (34.8 to 25.7)	−68.1 (−67.1 to−69.9)	−67.2 (−66.3 to −68.7)	−71 (−69.6 to −73.6)
Stroke	27.06 (29.17 to 23.00)	30.01 (31.63 to 27.00)	24.41 (26.86 to 19.78)	−68.3 (−67 to −70.2)	−68.8 (−67.7 to −70.2)	−68.7 (−67.1 to −71.4)
Ischemic stroke	16.72 (18.20 to 13.85)	18.07 (19.18 to 16.01)	15.42 (17.18 to 12.10)	−73.4 (−72.2 to −75.4)	−74.5 (−73.5 to −75.9)	−73.3 (−71.8 to −76)
Hypertensive heart disease	8.21 (9.07 to 6.66)	7.27 (7.75 to 6.40)	8.51 (9.56 to 6.60)	−5.1 (0.2 to −14.6)	−3.6 (1.5 to −10.5)	−5.1 (1.2 to −16.9)
Intracerebral hemorrhage	8.06 (8.58 to 7.08)	9.66 (10.14 to 8.89)	6.69 (7.30 to 5.58)	−56.9 (−54.7 to −59.8)	−56.2 (−54 to −58.7)	−58.4 (−55.6 to −62.2)
Arterial fibrillation	5.52 (6.04 to 4.56)	5.75 (6.10 to 5.05)	5.32 (5.93 to 4.20)	1.8 (6.1 to −4.6)	7.2 (10.7 to 1.9)	−1.1 (4.4 to −9.7)
Nonrheumatic valve disease	5.03 (5.48 to 4.21)	5.44 (5.78 to 4.80)	4.64 (5.18 to 3.71)	−5.9 (−0.2 to −14.4)	−0.7 (4.5 to −8.3)	−9.1 (−2.3 to −19.4)
Nonrheumatic calcification of aortic valve	4.23 (4.60 to 3.53)	4.68 (4.98 to 4.11)	3.82 (4.27 to 3.04)	6.6 (12.8 to −3.1)	8.2 (14.1 to −0.4)	6 (13.4 to −6)
Cardiomyopathy	3.57 (3.84 to 3.16)	5.08 (5.48 to 4.56)	2.31 (2.56 to 1.89)	−67.5 (−65.6 to −69.5)	−62 (−59.3 to −65)	−74.9 (−72.8 to −76.3)
Other cardiovascular disease	2.82 (3.02 to 2.49)	3.09 (3.26 to 2.85)	2.56 (2.81 to 2.18)	−51.6 (−49.3 to −54)	−54.9 (−52.8 to −57)	−50.2 (−47 to −53.7)
Other cardiomyopathy	2.78 (3.01 to 2.43)	3.72 (4.02 to 3.32)	2.02 (2.25 to 1.64)	−68.5 (−66.3 to −70.6)	−63.6 (−61.1 to −66.7)	−74.1 (−71.7 to −75.6)
Aortic aneurysm	2.57 (2.71 to 2.30)	3.95 (4.13 to 3.63)	1.50 (1.68 to 1.27)	−46.2 (−44.2 to −48.9)	−53.4 (−51.6 to −56.1)	−38.2 (−32.9 to −43.2)
**Subarachnoid hemorrhage**	2.28 (2.41 to 2.07)	2.28 (2.39 to 2.14)	2.29 (2.45 to 2.05)	−40.1 (−37.3 to −43.4)	−31.1 (−27.2 to −35.7)	−44.4 (−41.5 to −48.2)
Pulmonary arterial hypertension	0.18 (0.19 to 0.16)	0.14 (0.15 to 0.13)	0.21 (0.23 to 0.18)	−24.2 (−14.8 to −33.9)	−39.3 (−33.9 to −49.5)	−13.8 (0.01 to −30)
Rheumatic heart disease	1.51 (1.65 to 1.27)	1.28 (1.37 to 1.16)	1.64 (1.81 to 1.35)	−48.5 (−44.9 to −53.5)	−40.7 (−36.9 to −45.1)	−51.8 (−47.8 to −57.3)
Endocarditis	1.62 (1.75 to 1.41)	1.83 (1.94 to 1.67)	1.42 (1.57 to 1.18)	76.7 (94 to 53.7)	80.5 (99.2 to 57)	74.2 (92.9 to 49.9)
Nonrheumatic degenerative mitral valve	0.78 (0.85 to 0.66)	0.74 (0.79 to 0.66)	0.79 (0.88 to 0.65)	−42.5 (−38.6 to −48)	−34.2 (−29.9 to −38.9)	−46.2 (−41.2 to −52.9)
Alcoholic cardiomyopathy	0.59 (0.64 to 0.54)	1.14 (1.24 to 1.02)	0.11 (0.12 to 0.10)	−61.5 (−56.3 to 66.9)	−53.8 (−46.6 to −61)	−86.4 (−83.3 to −88.4)
Myocarditis	0.20 (0.23 to 0.17)	0.22 (0.25 to 0.19)	0.18 (0.20 to 0.15)	−67 (−64.5 to −69.3)	−67.4 (−64.6 to −70.3)	−68.7 (−65.3 to −71.4)
Other nonrheumatic valve disorder	0.02 (0.03 to 0.02)	0.02 (0.03 to 0.02)	0.03 (0.03 to 0.02)	−3.5 (11.5 to −16.6)	−17.5 (−2.2 to −31.6)	7.2 (27.5 to −11.2)
**Neoplasms**	125.8 (131.4 to 115.3)	159.5 (165.4 to 148.4)	100.01 (105.6 to 89.26)	−28.27 (−26.37 to −31.37)	−33.90 (−32.21 to −36.39)	−24.71 (−22.48 to −28.69)
Lung cancer	26.4 (27.69 to 24.47)	37.62 (39.46 to 35.08)	17.08 (18.18 to 15.45)	−26.41 (−23.23 to −30.08)	−44.25 (−41.55 to −47)	31.47 (37.23 to 24.47)
Colorectal cancer	15.1 (16.25 to 13.53)	19.22 (20.47 to 17.72)	11.83 (12.90 to 10.13)	−34.23 (−30.12 to −38.48)	−32.10 (−27.73 to −36.47)	−39.26 (−35.13 to −44.03)
Breast cancer	9.93 (10.57 to 8.82)	0.25 (0.27 to 0.22)	18.01 (19.11 to 16.15)	−40.35 (−37.62 to −44.01)	8.88 (18.62 to −0.86)	−38.15 (−35.38 to −41.88)
Prostate cancer	7.47 (8.04 to 6.68)	18.36 (19.81 to 16.26)	—^c^	−27.34 (−22.59 to −32.54)	−37.90 (−33.85 to −42.35)	—
Pancreatic cancer	9.26 (9.84 to 8.43)	10.62 (11.24 to 9.87)	8.02 (8.74 to 7.06)	8.61 (12.68 to 3.92)	4.30 (8.58 to 0.27)	11.51 (17.60 to 4.71)
Stomach cancer	5.57 (5.90 to 5.02)	7.71 (8.22 to 7.13)	3.83 (4.11 to 3.29)	−61.60 (−59.81 to −63.52)	−62.63 (−60.32 to −64.80)	−63.06 (−61.36 to −65.59)
Bladder cancer	4.29 (4.58 to 3.87)	7.71 (8.23 to 6.99)	1.83 (1.99 to 1.55)	−29.52 (−26.60 to −33.26)	−35.11 (−32.17 to −38.63)	−30.34 (−26.50 to −35.83)
Leukemia	4.53 (4.80 to 4.14)	5.94 (6.29 to 5.49)	3.44 (3.72 to 3.05)	−25.09 (−22.30 to −29.01)	−25.41 (−22.49 to −29.17)	−28.25 (−23.27 to −33.04)
Other malignant	2.81 (3.02 to 2.55)	2.92 (3.15 to 2.69)	2.72 (2.93 to 2.39)	−38.44 (−33.86 to −42.99)	−50.30 (−45.88 to −54.46)	−23.55 (−19.02 to −28.81)
Liver cancer	4.58 (4.84 to 4.23)	6.85 (7.30 to 6.38)	2.63 (2.81 to 2.33)	29.88 (36.84 to 22.96)	24.85 (34.45 to 15.46)	28.83 (34.79 to 21.26)
Non-Hodgkin lymphoma	3.77 (4.00 to 3.40)	4.82 (5.12 to 4.46)	2.91 (3.13 to 2.52)	−17.20 (−13.36 to −21.76)	−16.64 (−12.42 to −21.70)	−19.66 (−15.28 to −25.97)
Esophageal cancer	3.65 (3.81 to 3.41)	6.00 (6.25 to 5.66)	1.62 (1.74 to 1.44)	−24 (−21.34 to −27.03)	−28.19 (−25.26 to −31.14)	−20.94 (−16.93 to −25.06)
Kidney cancer	3.28 (3.50 to 3.01)	4.89 (5.19 to 4.52)	1.98 (2.16 to 1.74)	−10.46 (−4.76 to −16.53)	−10.39 (−4.78 to −16.57)	−17.45 (−11.20 to −23.84)
Brain and central nervous system cancer	4.34 (4.50 to 4.14)	5.37 (5.59 to 5.13)	3.40 (3.55 to 3.14)	0.16 (3.17 to −3.08)	1.34 (5.47 to −2.68)	−3.37 (0.17 to −8.48)
Ovarian cancer	3.00 (3.20 to 2.70)	—	5.55 (5.90 to 5.03)	−36.65 (−33.88 to −40.12)	—	−33.17 (−30.31 to −36.84)
Multiple myeloma	2.59 (2.76 to 2.33)	3.24 (3.45 to 2.96)	2.09 (2.26 to 1.81)	0.62 (5.80 to −5.99)	2.36 (8.62 to −5.50)	−4.79 (0.69 to −12.78)
Acute myeloid leukemia	2.43 (2.56 to 2.23)	3.04 (3.19 to 2.83)	1.94 (2.09 to 1.74)	16.98 (22.17 to 10.42)	21.01 (27.21 to 14.39)	8.38 (16.57 to −0.42)
Other neoplasms	1.52 (1.67 to 1.31)	2.09 (2.27 to 1.85)	1.12 (1.26 to 0.92)	37.11 (65.60 to 4.81)	36.01 (66.04 to 4.31)	28.82 (56.94 to −3.15)
Eye cancer	0.13 (0.14 to 0.11)	0.14 (0.16 to 0.13)	0.11 (0.13 to 0.10)	−15.42 (−8.86 to −22.30)	−17.46 (−11.66 to −24.92)	−15.28 (−7.18 to −22.97)
Gallbladder and biliary tract cancer	1.55 (1.66 to 1.39)	1.61 (1.72 to 1.47)	1.50 (1.62 to 1.31)	−49.55 (−47.57 to −51.92)	−34.72 (−31.83 to −38.38)	−56.69 (−54.54 to −59.46)
Malignant skin melanoma	1.74 (1.82 to 1.61)	2.23 (2.36 to 2.09)	1.33 (1.41 to 1.21)	3.50 (7.25 to −1.27)	13.06 (19.63 to 6.84)	−8.68 (−5.38 to −13.71)
Lip and oral cavity cancer	1.56 (1.64 to 1.44)	2.30 (2.44 to 2.14)	0.90 (0.96 to 0.80)	−32.75 (−29.07 to −36.53)	−43.59 (−39.77 to −47.31)	−0.03 (4.18 to −5.65)
Uterine cancer	1.39 (1.49 to 1.22)	—	2.53 (2.71 to 2.23)	−13.93 (−8.66 to −19.78)	—	−6.77 (−1.18 to −13.09)
Other leukemia	0.45 (0.49 to 0.39)	0.65 (0.72 to 0.55)	0.30 (0.34 to 0.26)	−18.09 (−12.46 to −24.24)	−15.85 (−9.14 to −23.46)	−27.23 (−19.51 to −34.90)
Cervical cancer	1.22 (1.30 to 1.12)	—	2.29 (2.43 to 2.11)	−53.77 (−51.46 to −56.22)	—	−51.20 (−48.74 to −53.78)
Mesothelioma	0.99 (1.05 to 0.91)	1.78 (1.88 to 1.65)	0.36 (0.39 to 0.32)	−8.14 (5.06 to −19.92)	−11.56 (4.23 to −23.60)	−16.91 (−4.68 to −25.74)
Chronic lymphoid leukemia	0.95 (1.04 to 0.82)	1.36 (1.49 to 1.20)	0.65 (0.73 to 0.53)	−33.73 (−28.77 to −39.45)	−36.97 (−32.16 to −42.40)	−34.09 (−27.18 to −41.63)
Larynx cancer	0.99 (1.05 to 0.92)	1.90 (2.02 to 1.76)	0.23 (0.25 to 0.21)	−58.22 (−55.60 to −60.85)	−62.85 (−60.45 to −65.22)	−26.91 (−21.93 to −32.20)
Other pharynx cancer	1.10 (1.21 to 1.00)	1.89 (2.08 to 1.72)	0.39 (0.43 to 0.35)	−17.63 (−10.62 to −24.47)	−26.26 (−19.31 to −32.75)	32.01 (44.25 to 21.40)
Nonmelanoma skin cancer	0.64 (0.68 to 0.56)	0.98 (1.04 to 0.88)	0.40 (0.44 to 0.33)	−1.83 (2.08 to −6.68)	2.59 (6.86 to −2.54)	−14.33 (−10.02 to −20.41)
Thyroid cancer	0.39 (0.42 to 0.34)	0.40 (0.43 to 0.36)	0.37 (0.41 to 0.32)	−42.80 (−38.14 to −47.16)	−29.53 (−23.59 to −34.96)	−49.52 (−45.31 to −53.52)
Chronic myeloid leukemia	0.29 (0.32 to 0.25)	0.38 (0.43 to 0.33)	0.22 (0.25 to 0.18)	−74.58 (−72.04 to −76.92)	−74.38 (−71.72 to −77.10)	−75.84 (−71.59 to −79.34)
Hodgkin lymphoma	0.28 (0.31 to 0.26)	0.36 (0.39 to 0.33)	0.21 (0.23 to 0.19)	−60.01 (−57.05 to −62.65)	−60.11 (−57.24 to −62.74)	−60.61 (−57.42 to −63.56)
Acute lymphoid leukemia	0.42 (0.44 to 0.40)	0.51 (0.54 to 0.48)	0.34 (0.36 to 0.31)	−51.20 (−48.53 to −53.68)	−50.33 (−47.57 to −53.28)	−52.54 (−48.92 to −56.02)
Nasopharynx cancer	0.24 (0.26 to 0.21)	0.36 (0.41 to 0.33)	0.12 (0.13 to 0.11)	−51.99 (−46.09 to −56.73)	−55.82 (−50.21 to −60.26)	−42.47 (−35.66 to −48.09)
Testicular cancer	0.12 (0.13 to 0.11)	0.25 (0.27 to 0.24)	—	−51.83 (−48.36 to −54.71)	−53 (−49.59 to −55.76)	—

a UI: uncertainty interval. UI shows the highest to lowest interval. All estimate values are significant as UI does not contain 0 value.

b Values are reported to 1 or 2 decimal places based on the precision of the estimates.

c Not applicable.

### Neoplasm Statistics

Neoplasms (cancers) accounted for approximately 27.1% (95% UI 28.6%-24.3%) of total deaths in Western Europe. The age-standardized death rate for neoplasms in the region was 125.8 per 100,000 population (95% UI 131.4-115.3) in the same year (Tables1  2). The 3 cancer subtypes with the highest death burden in Western Europe in 2021 were lung cancer, colorectal cancer, and breast cancer (Table 2). The death burden of neoplasms in Western Europe has also shown a downward trend from 1990 to 2021. The age-standardized death rate of neoplasms in the region had a decrease of –28.27 (95% UI −26.37 to −31.37) during this period. This decrease was particularly evident for the 3 leading cancer subtypes: lung cancer (–26.41%, 95% UI −23.23% to −30.08%), colorectal cancer (–34.23%, 95% UI −30.12% to −38.48%), and breast cancer (–40.35%, 95% UI −37.62% to −44.01%) (Table 2).

### 2021 Disease Burden of CVDs and Neoplasms by Country in Western Europe

**Figure 2.**
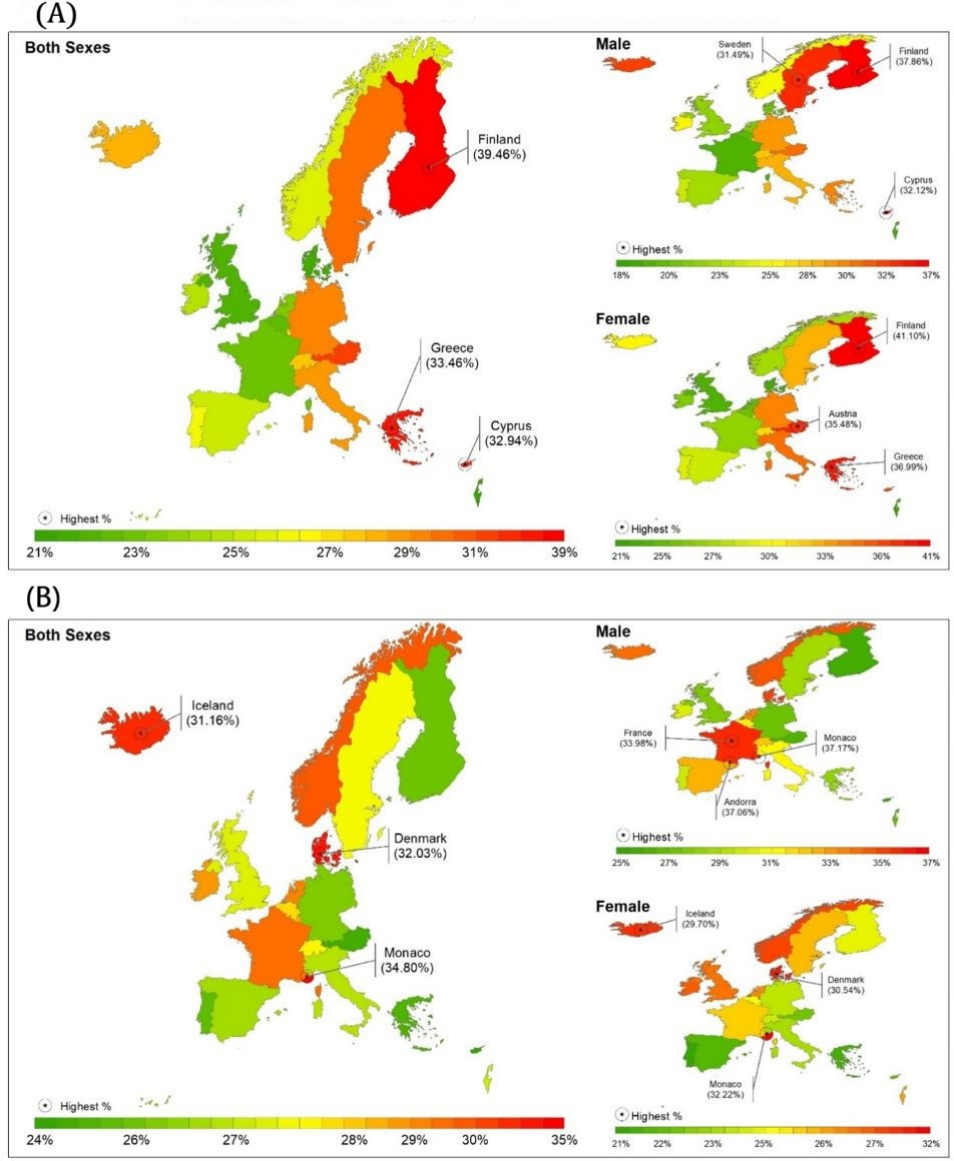
(A) Cardiovascular diseases as a percentage of total deaths in 2021 across all ages. (B) Neoplasms as a percentage of total deaths in 2021 across all ages. Both are shown for both sexes and by gender for Western European countries.

In 2021, Finland, Greece, and Cyprus showed the highest burden of CVDs across Western Europe, accounting for 39.5% (95% UI 42.7%-33.0%), 33.5% (95% UI 35.6%-29.0%), and 32.9% (95% UI 35.6%-29.3%) of total deaths, respectively (Figure 2A). Meanwhile, Monaco, Denmark, and Iceland ranked highest for neoplasm-related deaths in Western Europe, with proportions of 34.8% (95% UI 38.9%-30.2%), 32.0% (95% UI 33.8%-29.3%), and 31.2% (95% UI 33.1%-27.9%) of total deaths, respectively (Figure 2B).

### Distribution of Disease Burden of CVDs and Neoplasms by Sex in Western Europe

**Figure 3.**
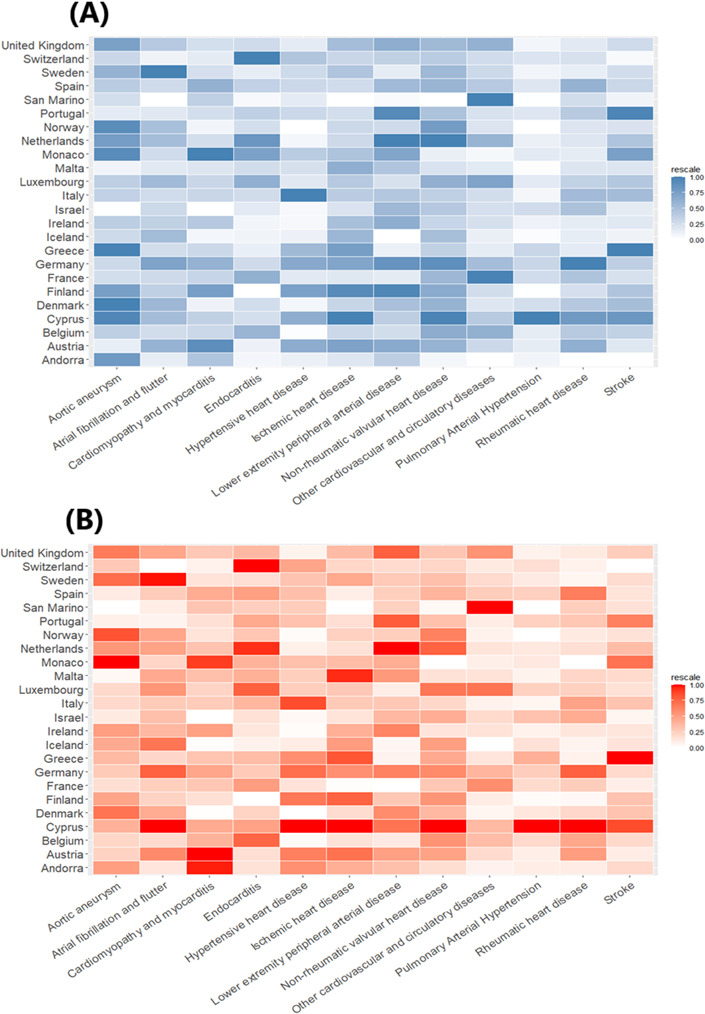
Age-standardized death rates of cardiovascular diseases in 2021 by country in Western Europe, showing data for (A) males and (B) females. The age-standardized death rates were calculated using the world standard population as evaluated by the Global Burden of Diseases 2021 study.

**Figure 4.**
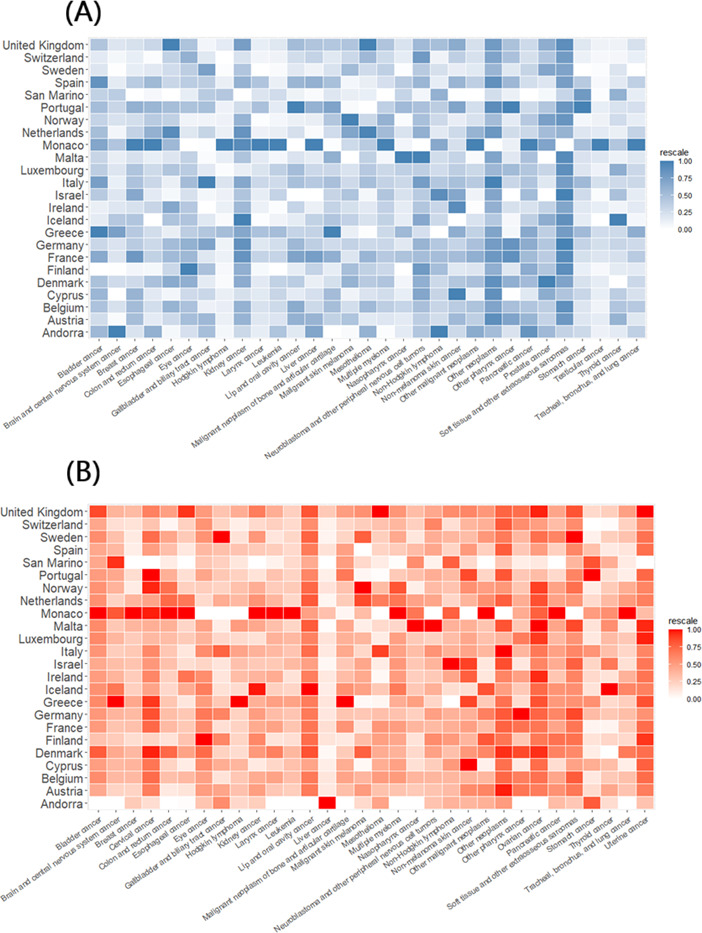
Age-standardized death rates of neoplasms by country in Western Europe in 2021, showing data for (A) males and (B) females. The age-standardized death rates were calculated using the world standard population as evaluated by the Global Burden of Diseases 2021 study.

In 2021, males in Western Europe experienced a higher death burden from CVDs, with an age-standardized death rate of 132.4 per 100,000 (95% UI 138.8-120.1) compared to females who had a rate of 85.4 per 100,000 (95% UI 93.9-68.9). Across almost all Western European countries, death rates due to CVDs were higher for males than females (Figure 3). Similarly, males faced a greater death burden from neoplasms in Western Europe in 2021, with an age-standardized death rate of 159.5 per 100,000 (95% UI 165.4-148.4) compared to females at 100.0 per 100,000 (95% UI 105.6-89.26). Across all 24 Western European countries, males consistently had higher death rates for neoplasms than females (Figure 4).

### Distribution of Disease Burden of CVDs and Neoplasms by Age in Western Europe

**Figure 5.**
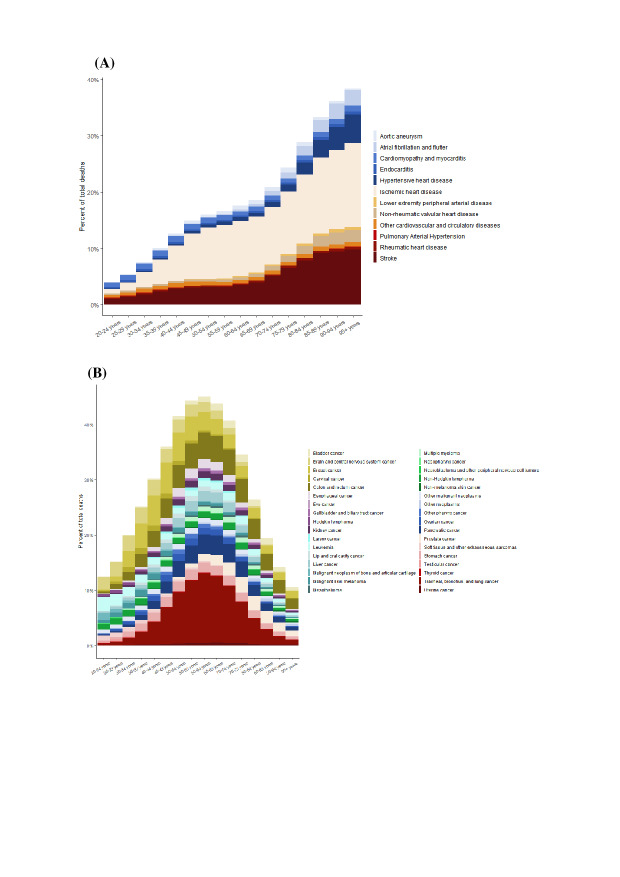
Death burden in 2021 by age group and cause for both sexes in Western Europe as a percentage of total deaths from (A) cardiovascular diseases and (B) neoplasms.

In 2021, the burden of CVD deaths increased significantly with advancing age, with older age groups experiencing higher mortality rates. The primary contributors to the CVD death burden included aortic aneurysm; atrial fibrillation and flutter; cardiomyopathy and myocarditis; endocarditis; and hypertensive heart disease (Figure 5A). The analysis also examined the age distribution of subtypes within specific cardiovascular conditions, including stroke, nonrheumatic valvular heart disease, and cardiomyopathy. The age distribution patterns for stroke subtypes and nonrheumatic valve disease closely resembled the overall trend observed for CVDs. In contrast, the different subgroups of cardiomyopathy displayed distinct age-related characteristics (Figure 5A).

Figure 5B highlights the distribution of cancer-related deaths across different age groups in Western Europe in 2021, illustrating how the type and burden of cancers change significantly with age. Lung, breast, prostate, and colorectal cancers are the most prevalent in the older population. Specifically, the peak in the 60-65 years age group is primarily due to bladder cancer, brain and central nervous system cancer, breast cancer, cervical cancer, colorectal cancer, and other age-related cancers. The data show that, as individuals age, the prevalence of certain cancers also increases.

### The Association Between Covariates and Age-Standardized Death and DALY Rate by Sex in 2021

**Figure 6.**
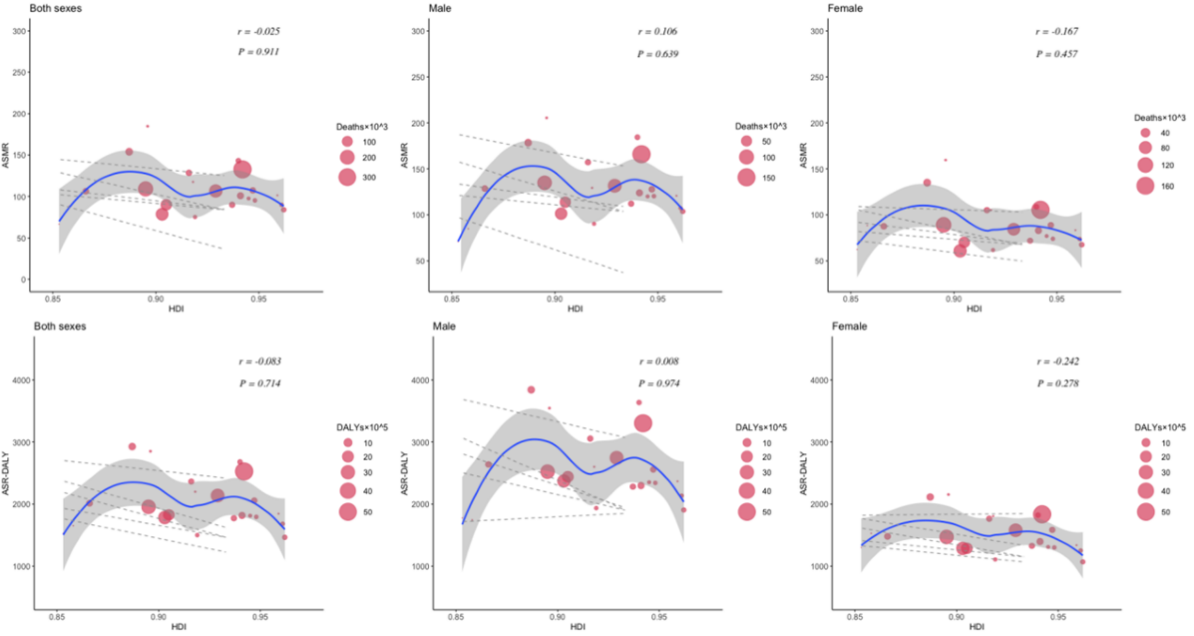
The correlation between the HDI and age-standardized deaths and DALYs of CVDs in Western European countries in 2021 by sex. Circle size represents the number of death cases and DALYs associated with CVDs. The blue line and gray shadows represent the overall trend and 95% CIs in age-standardized rates associated with HDI, and the dashed lines represent quantile regression estimated fit (95th, 75th, 50th, 25th, and 5th percentiles). The *r* indices and *P* values presented were derived from Pearson correlation analysis. ASMR: age-standardized mortality rate; ASR: age-standardized rate; CVD: cardiovascular disease; DALY: disability-adjusted life years; HDI: Human Development Index.

**Figure 7.**
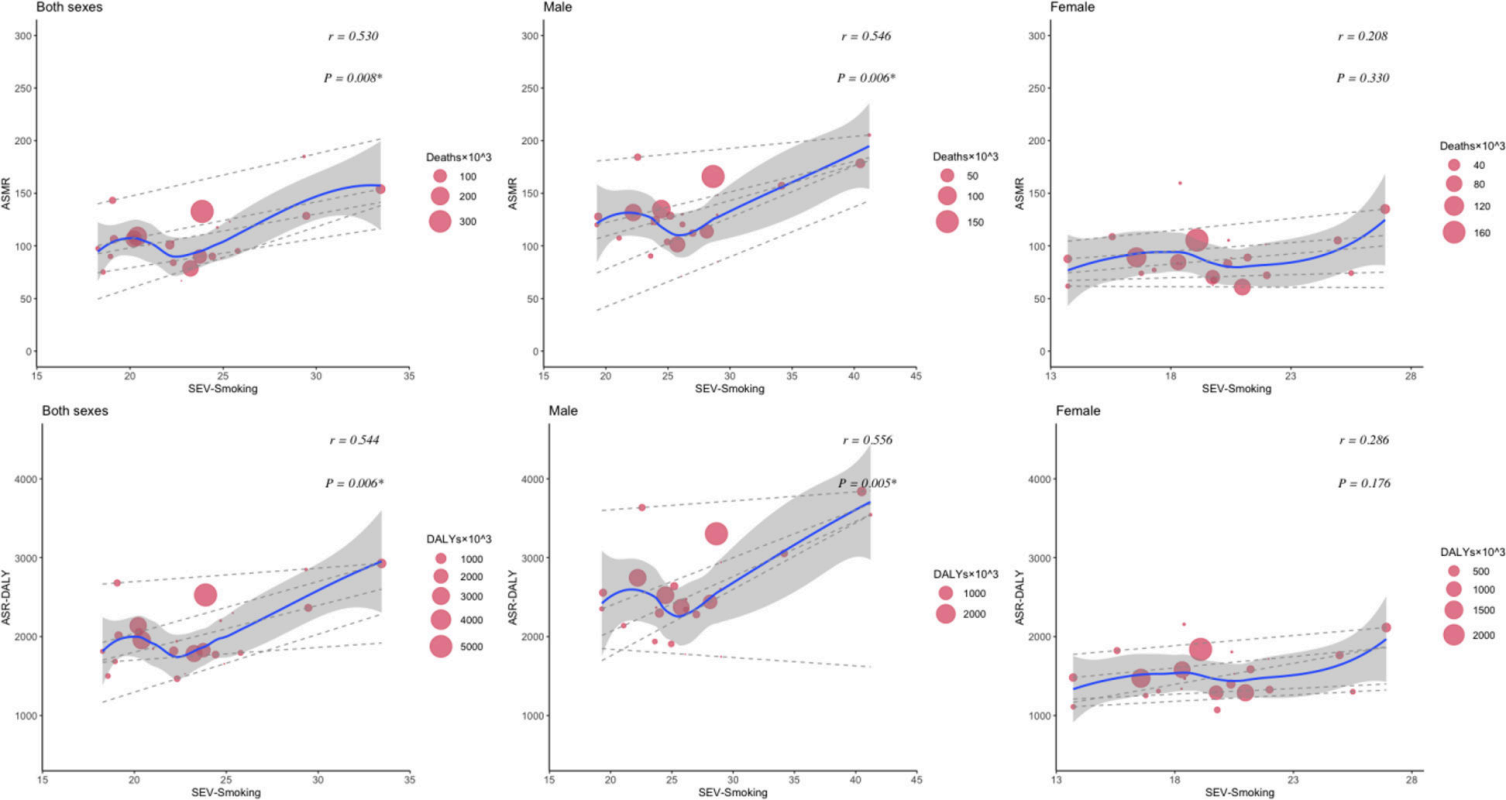
The correlation between SEV-Smoking and age-standardized mortality and DALYs of CVDs in Western European countries in 2021 by sex. Circle size represents the number of death cases and DALYs associated with CVDs. The blue line and gray shadows represent the overall trend and 95% CIs in age-standardized rates associated with SEV-Smoking, and the dashed lines represent quantile regression estimated fit (95th, 75th, 50th, 25th, and 5th percentiles). The *r* indices and *P* values presented were derived from Pearson correlation analysis. An asterisk (*) indicates a significant correlation coefficient between two variables. ASMR: age-standardized mortality rate; ASR: age-standardized rate; CVD: cardiovascular disease; DALY: disability-adjusted life years; SEV-Smoking: summary exposure value of smoking.

**Figure 8.**
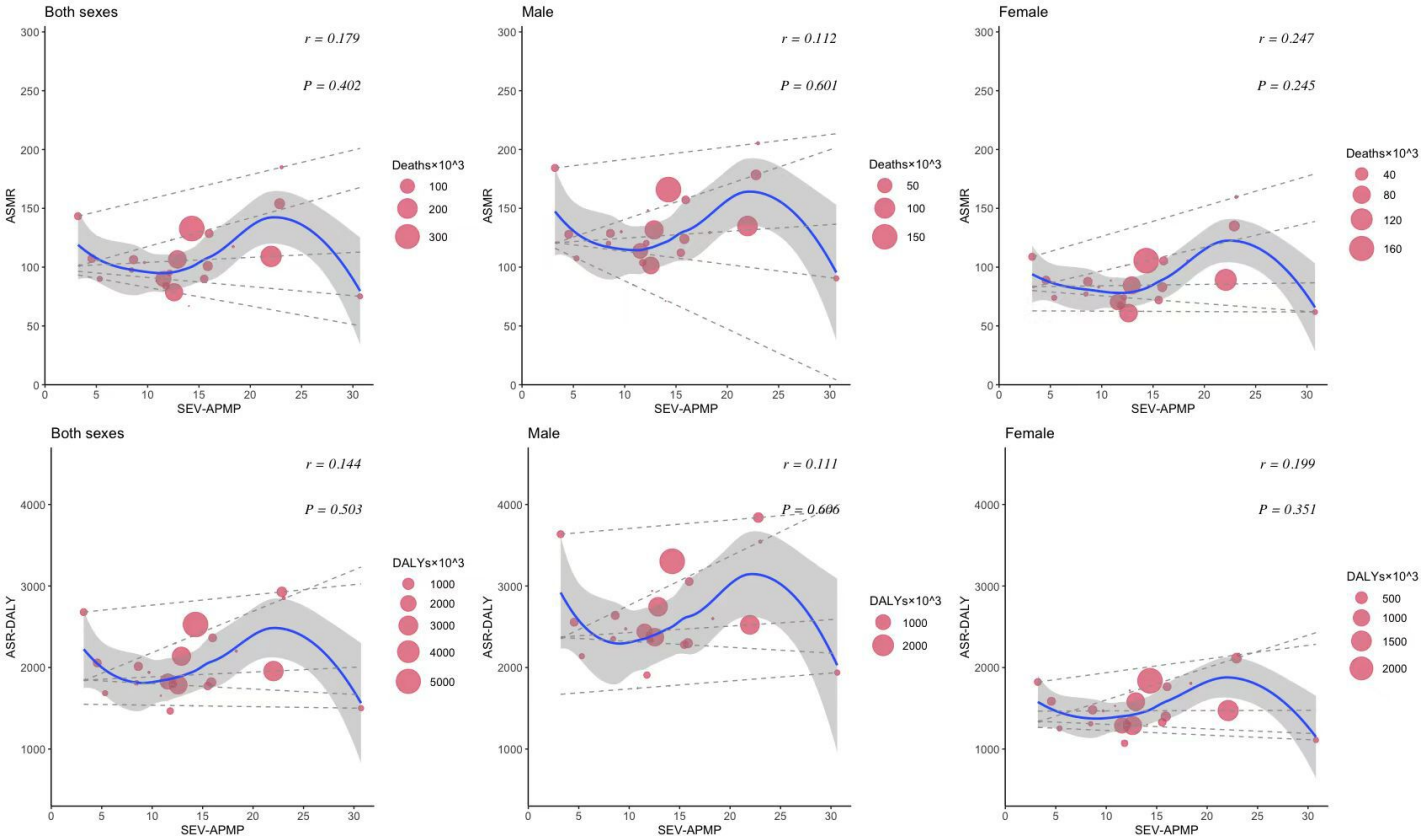
The correlation between SEV-APMP and age-standardized mortality and DALYs of CVDs in Western European countries in 2021 by sex. Circle size represents the number of death cases and DALYs associated with CVDs. The blue line and gray shadows represent the overall trend and 95% CIs in age-standardized rates associated with SEV-APMP, and the dashed lines represent quantile regression estimated fit (95th, 75th, 50th, 25th, and 5th percentiles). The *r* indices and *P* values presented were derived from Pearson correlation analysis. ASMR: age-standardized mortality rate; ASR: age-standardized rate; CVD: cardiovascular disease; DALY: disability-adjusted life years; SEV-APMP: summary exposure value of ambient particulate matter pollution.

The relationship between the HDI and the burden of CVDs and neoplasms revealed distinct patterns. For CVDs, age-standardized deaths and DALYs initially increased and then decreased as HDI improved. In Western European countries with an HDI of approximately 0.95, both age-standardized deaths and DALY cases reached their highest levels. Although there was an insignificant correlation (*P*=.9 for death, *P*=.7 for DALYs) between age-standardized CVD death and DALY rates with increased HDI, high CVD cases were observed in both males and females (Figure 6). Notably, a significant positive correlation was found between SEV-Smoking and age-standardized rates of CVD death (*r*=0.55, *P*=.006) and DALYs (*r*=0.56, *P*=.005) among males in Western Europe in 2021 (Figure 7). The results suggest that SEV-Smoking has a stronger impact on both age-standardized CVD death rates and DALYs in males compared to females. These interpretations highlight the gender differences in the impact of smoking on cardiovascular health, with smoking being a more prominent risk factor for males (Figure 7). No significant correlation was found between age-standardized death rates or DALY rates of CVDs and SEV-APMP, with all *P* values exceeding .05 (Figure 8).

**Figure 9.**
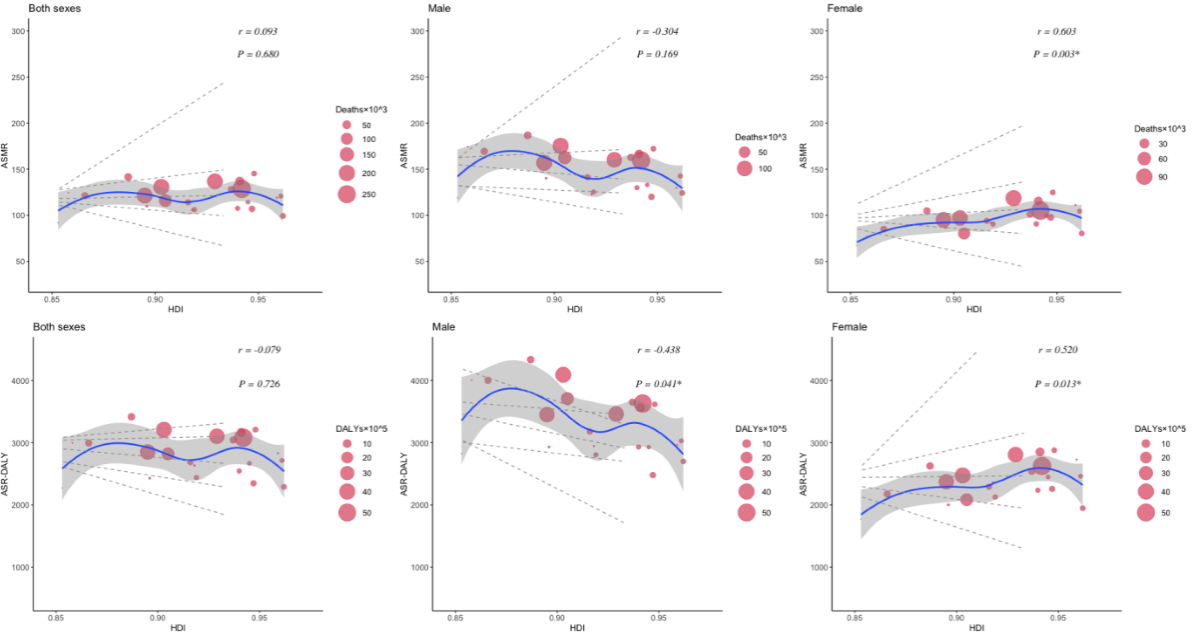
The correlation between the HDI and age-standardized mortality and DALYs of neoplasms in Western European countries in 2021 by sex. Circle size represents the number of death cases and DALYs associated with neoplasms. The blue line and gray shadows represent the overall trend and 95% CIs in age-standardized rates associated with HDI, and the dashed lines represent quantile regression estimated fit (95th, 75th, 50th, 25th, and 5th percentiles). The *r* indices and *P* values presented were derived from Pearson correlation analysis. An asterisk (*) indicates a significant correlation coefficient between two variables. ASMR: age-standardized mortality rate; ASR: age-standardized rate; CVD: cardiovascular disease; DALY: disability-adjusted life years; HDI: Human Development Index.

**Figure 10.**
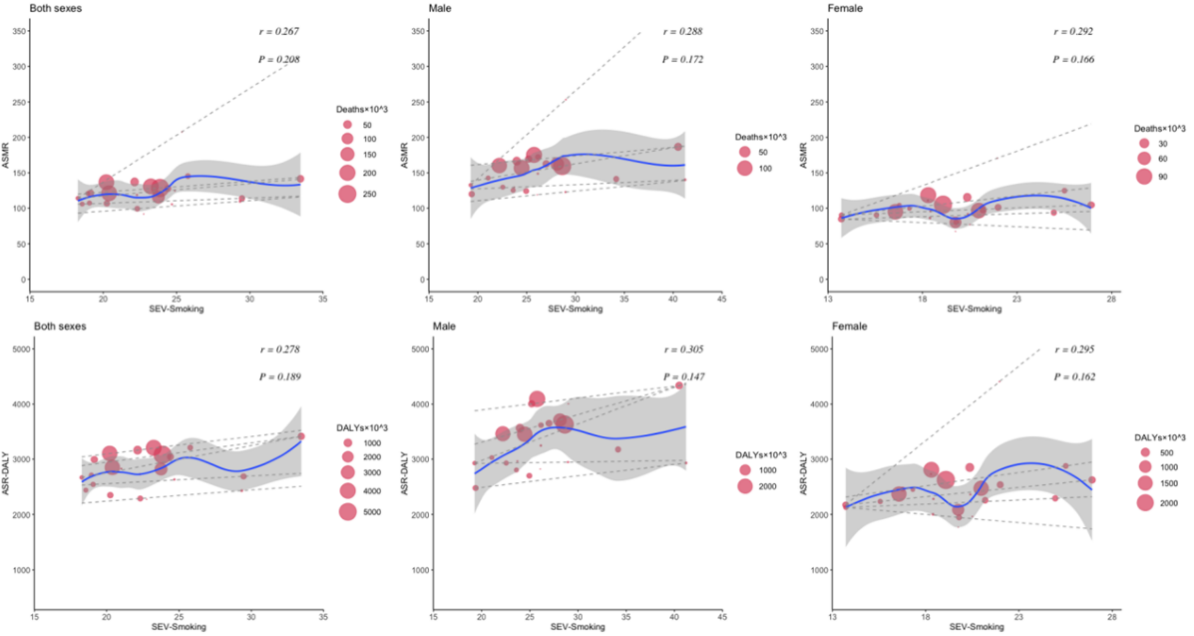
The correlation between SEV-Smoking and age-standardized mortality and DALYs of neoplasms in Western European countries in 2021 by sex. Circle size represents the number of death cases and DALYs associated with neoplasms. The blue line and gray shadows represent the overall trend and 95% CIs in age-standardized rates associated with SEV-Smoking, and the dashed lines represent quantile regression estimated fit (95th, 75th, 50th, 25th, and 5th percentiles). The *r* indices and *P* values presented were derived from Pearson correlation analysis. ASMR: age-standardized mortality rate; ASR: age-standardized rate; DALY: disability-adjusted life years; SEV-Smoking: summary exposure value of smoking.

The relationship between HDI and neoplasm outcomes shows marked differences between males and females. Higher HDI was associated with increased neoplasm death rates (*r*=0.60, *P*=.003) and DALYs (*r*=0.52, *P*=.013) in females, while in males, higher HDI correlated with decreased DALYs (*r*=−0.44, *P*=.04), though the death rates show a nonsignificant increase (Figure 9). No significant association was observed between the age-standardized death and DALY rates of neoplasms and SEV-Smoking or SEV-APMP (Figures10  11).

**Figure 11.**
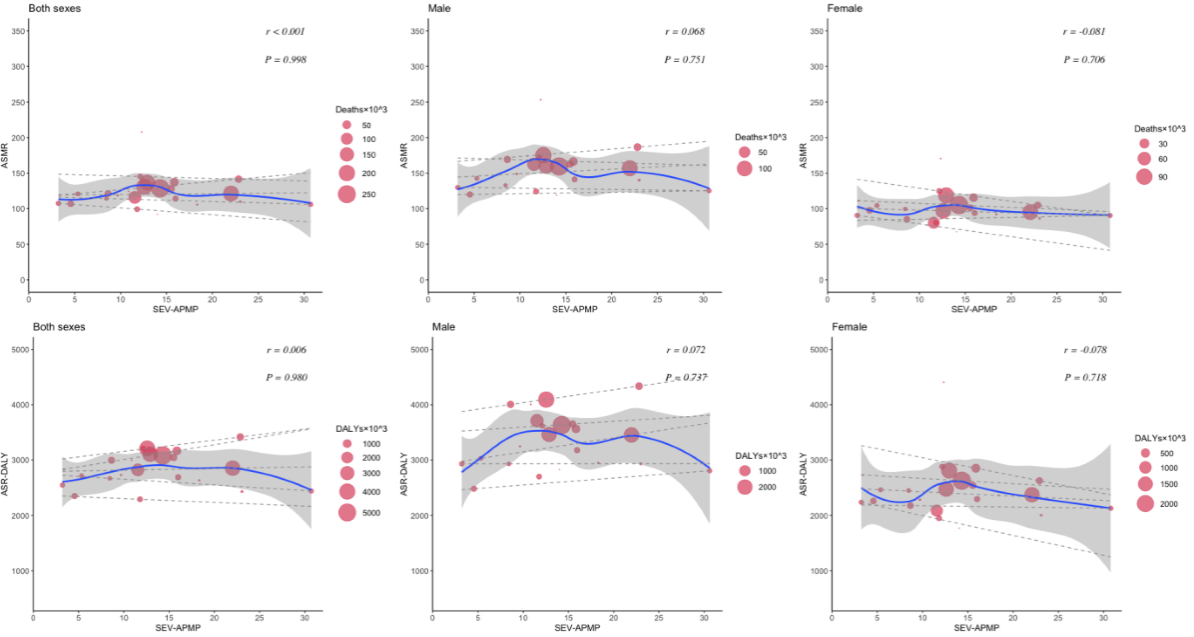
The correlation between SEV-APMP and age-standardized mortality and DALYs of neoplasms in Western European countries in 2021 by sex. Circle size represents the number of death cases and DALYs associated with neoplasms. The blue line and gray shadows represent the overall trend and 95% CIs in age-standardized rates associated with SEV-APMP, and the dashed lines represent quantile regression estimated fit (95th, 75th, 50th, 25th, and 5th percentiles). The *r* indices and *P* values presented were derived from Pearson correlation analysis. ASMR: age-standardized mortality rate; ASR: age-standardized rate; DALY: disability-adjusted life years; SEV-APMP: summary exposure value of ambient particulate matter pollution.

## Discussion

### Principal Findings and Comparisons With Prior Work

The findings of this study yielded valuable insights into the distribution, risk factors, and death burden associated with CVDs and neoplasms in Western Europe from 1990 to 2021. The results underscored the substantial impact of these NCDs on the population of the region, emphasizing the urgent need to address these health challenges in alignment with the goals outlined in the WHO’s Action Plan. The study’s comprehensive analysis sheds light on the prevailing trends and patterns, providing a foundation for targeted interventions and policy initiatives aimed at reducing the burden of CVDs and neoplasms and improving public health outcomes in Western Europe. Moreover, this study provided key clinical implications to strengthen CVD prevention strategies, with a focus on early detection and risk factor management for ischemic heart disease and stroke. In addition, cancer screening and early detection programs should be enhanced, particularly for lung and colorectal cancer, while approaches should be tailored to address disparities. Targeted interventions should be implemented to address modifiable risk factors, such as smoking, and tackle socioeconomic inequities. Robust monitoring and evaluation systems should be established to assess long-term impacts and inform the development of effective strategies.

In 2021, following the COVID-19 pandemic, CVDs and neoplasms were the leading causes of death across all age groups in every Western European country. These NCDs accounted for the highest death rates and represented the primary contributors to the burden of disease in the region. A similar trend was observed in 2019 in this region [15]. Moreover, a notable shift was observed in Western Europe, with the majority of countries experiencing the highest age-standardized death rates for neoplasms, surpassing CVDs as the leading cause of death. This pattern held true for both males and females in these countries. This finding represents a deviation from the global epidemiological trend, where CVDs traditionally hold the top position, followed by cancer as the second highest cause of death [16]. Furthermore, findings of a recent study indicated a potential shift in which cancer could become the primary cause of premature death in most countries in the coming decades [17,18]. As a result, it is essential for governments to consider these transitions when developing cancer policies tailored to the disease profiles of their respective regions.

The overall data indicated that the death burden of CVDs in Western Europe showed a downward trend from 1990 to 2021. The age-standardized death rate of CVDs in the region decreased by 61.9% during this period. This observed decline in the CVD mortality burden in Western Europe aligns with the global epidemiological trend reported in 2019, which also showed a decrease in the age-standardized death rate for CVDs worldwide [16]. Similar to the trends observed for CVDs, the death burden of neoplasms in Western Europe also showed a downward trajectory from 1990 to 2021. The age-standardized death rate of neoplasms in the region decreased by 28.27% during this period. However, this decline in the cancer mortality burden in Western Europe is not in alignment with the global epidemiological pattern, as various researchers have estimated that the number of new cancer cases is increasing worldwide, leading to an overall increase in the global cancer death burden [19,20].

In 2021, Finland, Greece, and Cyprus had the highest burden of CVDs among Western European countries. On the other hand, Monaco, Denmark, and Iceland had the highest rates of neoplasm-related deaths in the region. These results indicate that, despite overall advancements in health, health disparities related to NCDs still persist in several European countries [21]. These disparities often reflect underlying socioeconomic inequalities, with wealthier countries generally experiencing better health outcomes. To address these disparities, efforts should focus on improving educational opportunities, promoting fair income distribution, encouraging healthy behaviors, and ensuring equitable access to health care services [1,22]. In the majority of Western European countries, there was a consistent pattern of higher death rates from CVDs among males compared to females. This indicates that males are more vulnerable to CVD-related mortality in these regions. This disparity in CVD mortality between genders may be attributed to higher prevalence rates of certain risk factors among men, including smoking, excessive alcohol consumption, unhealthy dietary habits, sedentary lifestyles, and lower levels of physical activity. These findings diverge from previous studies that have emphasized CVD as a significant cause of death in women [23-25]. In the case of neoplasms, the death burden of all neoplasm subtypes, except for a few cancers, was higher in males compared to females. This result is similar to a previous study where males had significantly higher incidence rates of cancer [26]. The higher cancer burden in males compared to females can be attributed to factors such as risk behaviors (eg, smoking, excessive alcohol consumption), hormonal and genetic differences, lower health care–seeking behavior, occupational exposures, and specific cancers related to male anatomy. Addressing these factors through targeted interventions and promoting healthier behaviors can help reduce the cancer burden in males [27-30].

The findings further revealed that in Western Europe, older populations faced a higher risk of mortality from both CVDs and various types of neoplasms compared to younger age groups. The increased mortality rate in the older population can be attributed to age-related changes, cumulative exposure to risk factors, existing health conditions, weakened immune systems, reduced resilience, and disparities in health care access. To mitigate these risks, it is essential to provide comprehensive geriatric care, implement preventive strategies, conduct regular health screenings, manage chronic diseases, and ensure equitable access to health care services. These measures are vital for promoting the well-being of the older population [31].

In this study, there was no significant correlation observed between age-standardized CVD death rates and increased HDI. However, when examining the relationship between HDI and neoplasm outcomes, the findings revealed that higher HDI was associated with increased neoplasm death rates and DALYs specifically among females in Western Europe. In other words, as the HDI increased, there was a higher prevalence of neoplasm-related mortality and disease burden among women. Global estimates indicate that this is particularly true for a few cancers rather than for all cancers. In the case of breast cancer, it is true. In countries with a very high HDI, 1 in 12 women will be diagnosed with breast cancer in their lifetime and 1 in 71 women will die from it. By contrast, in countries with a low HDI, 1 in 27 women will be diagnosed with breast cancer in their lifetime; however, 1 in 48 women will die from it [32]. Thus, these findings highlight the importance of addressing the specific challenges faced by females in relation to neoplastic diseases, especially in more developed regions. In addition, the study highlighted an important gender disparity in the impact of smoking on cardiovascular health. It revealed that SEV-Smoking has a stronger effect on age-standardized CVD death rates and DALYs in males compared to females. Previous studies also suggested that the impact of smoking on CVD mortality and disease burden is more pronounced in males compared to females [33,34].

Overall, the study findings highlighted the significant impact of CVDs and neoplasms in Western Europe, ranking as the leading causes of death. Although there has been some progress, the burden of these diseases remains high, indicating there are persistent challenges to achieving the goals set forth in the Action Plan for the Prevention and Control of NCDs in the WHO European Region 2016‐2025. Monitoring the progress of the Action Plan reveals both positive advancements and areas that require further attention. Continual monitoring and evaluation are crucial in identifying gaps and guiding evidence-based interventions. Successful reduction of the burden of CVDs and neoplasms and the achievement of the Action Plan’s objectives for a healthier population necessitate a collaborative approach involving policy makers, health care professionals, and communities. Moreover, to tackle the significant burden of CVDs and neoplasms in Western Europe, it is crucial to implement targeted interventions. These interventions should incorporate gender-specific approaches, considering the higher mortality rates among males and the elevated risk associated with advanced age. Additionally, addressing socioeconomic factors is essential in order to prioritize resources effectively and implement interventions that reduce health inequalities.

### Limitations

The study has several limitations that should be considered. First, the analysis relied on data obtained from GBD 2021, which may be subject to data limitations, reporting biases, and methodological variations across countries. Second, the study focused on Western Europe, and the findings may not be generalizable to other regions or countries with different demographics and health care systems. Third, the analysis primarily examined CVDs and neoplasms; other NCDs were not extensively explored. Fourth, the study relied on aggregated data; individual-level factors or contextual variables were not taken into account. Finally, although quantile regression provides insights into different segments of the population, it does not establish causality between the covariates and disease burden.

### Conclusion

In conclusion, this study revealed a significant burden of CVDs and neoplasms in Western Europe. The findings highlighted the need for targeted interventions to address risk factors and improve population health outcomes. Regional variations, gender differences, and the impact of socioeconomic factors underscored the complexity of these diseases. Furthermore, the study evaluated the progress made in implementing the Action Plan and its impact on improving health outcomes. By recognizing achievements and identifying areas that require further attention, this research provides valuable insights for policy makers and health care professionals. It emphasizes the need to continue advancing the Action Plan’s goals, ultimately leading to better prevention and control of NCDs in Western Europe.

## Abbreviations

APC: annual percent change
ASMR: age-standardized mortality rate
ASR: age-standardized rate
CVD: cardiovascular disease
DALY: disability-adjusted life year
GBD: Global Burden of Disease
NCD: noncommunicable disease
SEV-APMP: summary exposure value of ambient particulate matter pollution
SEV-Smoking: summary exposure value of smoking
UI: uncertainty interval
WHO: World Health Organization
